# Pneumatic Coiling Actuator Inspired by the Awns of *Erodium cicutarium*

**DOI:** 10.3389/frobt.2020.00017

**Published:** 2020-02-18

**Authors:** Ryan Geer, Steven Iannucci, Suyi Li

**Affiliations:** Department of Mechanical Engineering, Clemson University, Clemson, SC, United States

**Keywords:** plant motion, soft robotic actuator, tilted helix, coiling motion, reinforcing fiber

## Abstract

This study examines the coiling and uncoiling motions of a soft pneumatic actuator inspired by the awn tissue of *Erodium cicutarium*. These tissues have embedded cellulose fibers distributed in a *tilted* helical pattern, which induces hygroscopic coiling and uncoiling in response to the daily changes in ambient humidity. Such sophisticated motions can eventually “drill” the seed at the tip of awn tissue into the soil: a drill bit in the plant kingdom. Through finite element simulation and experimental testing, this study examines a soft pneumatic actuator that has a similar reinforcing fiber layout to the Erodium plant tissue. This actuator, in essence, is a thin-walled elastomeric cylinder covered by tilted helical Kevlar fibers. Upon internal pressurization, it can exhibit a coiling motion by a combination of simultaneous twisting, bending, and extension. Parametric analyses show that the coiling motion characteristics are directly related to the geometry of tilted helical fibers. Notably, a moderate tilt in the reinforcing helical fiber leads to many coils of small radius, while a significant tilt gives fewer coils of larger radius. The results of this study can offer guidelines for constructing plant-inspired robotic manipulators that can achieve complicated motions with simple designs.

## 1. Introduction

In our popular belief, plants are static and immobile, but this couldn't be further from the truth. Plants are capable of achieving many sophisticated motions—almost continuously—without any muscles or nerve systems (Burgert and Fratzl, 2009; Martone et al., 2010; Dumais and Forterre, 2012). These motions are central to plants' survival and fitness, and they vary drastically in terms of their actuation and control principle, physiological origin, magnitude, and speed. Some plant motions are reversible so they can serve as blueprints for engineering adaptive structures and robots (Forterre, 2013; Guo et al., 2015; Charpentier et al., 2017; Li and Wang, 2017). For example, the trap closing motion in Venus flytrap (*Dionaea muscipula*) is rapid enough to capture agile insect prey like the fruit flies, which are then digested as nutrition supplement (Forterre et al., 2005; Skotheim and Mahadevan, 2005). The Venus flytrap gains its speed from actively changing the turgor pressure in its motor cells and exploiting an embedded snap-through instability, and it has inspired many robotic grippers (Kim et al., 2014; Zhang et al., 2016; Wani et al., 2017) and adaptive cellular structures (Gramüller et al., 2015; Li and Wang, 2015b). On the other end of the speed spectrum is the pinecone opening motion. It is driven by tissue swelling and shrinking in response to the ambient humidity change (aka. hygroscopy), and a bimorph construction in the pinecone scales directs this swelling into bending to create the opening/closing motions (Dawson et al., 1997). The actuation principle and physiological features of pinecone have inspired new responsive materials (Erb et al., 2013; Wu et al., 2013; Wei et al., 2014; Sydney Gladman et al., 2016), and building envelopes (Menges and Reichert, 2012; Holstov et al., 2015). Besides these reversible motions, plants can also move slowly and irreversibly, such as the growth of roots and tendrils. These growth motions have recently inspired a new family of robots with unique navigation and exploration capabilities (Sadeghi et al., 2014, 2017; Hawkes et al., 2017; Nahar et al., 2017). Even to this day, we are still discovering new examples of plant motions and developing engineered systems based on the lessons from them.

**Figure 1:**
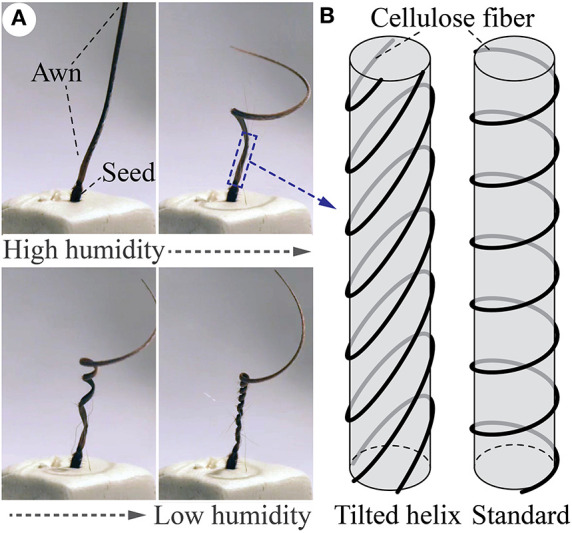
Coiling motions induced by the tilted helical reinforcing fibers. **(A)** When ambient humidity drops, the awn of *Erodium cicutarium* will coil (from upper left figure to lower right). Such coiling will reverse if the humidity increases. Figures adapted from Evangelista et al. (2011) with permission from the Company of Biologists Ltd. **(B)** The underlying tilted helical cellulose fiber provides the structural constraints required for this coiling motion. A standard helix is also illustrated for comparison.

In this study, we focus on a particularly intriguing plant motion: coiling in the seed awn of *Erodium Cicutarium* plant and its relatives (Stamp, 1984). The long and slender appendage tissues of their seed can coil and uncoil in response to the diurnal humidity cycle. Such a repetitive motion, combined with the angled bristles on the seed and along the side of the awn, can eventually bury the seed into the soil for germination (Evangelista et al., 2011) (Figure 1A). The key ingredient to achieving this coiling is in the plant cell walls. That is, the walls of the *Erodium* awn cells have reinforcing cellulose fibers arranged in a *tilted helical* pattern (Figure 1B) so that the longitudinal axis of the helical fibers does not align with the cell axis. As a result, when the ambient humidity drops, plant tissues in the *Erodium* awn would shrink in volume due to hygroscopy, and their tilted helical fibers can direct this shrinking into a coiling motion (Abraham et al., 2012; Aharoni et al., 2012; Abraham and Elbaum, 2013; Elbaum and Abraham, 2014; Jung et al., 2014; Zhao et al., 2017). Similarly, the awn would uncoil when humidity increases, essentially creating a “drill bit” in the plant kingdom.

While the role of tilted helical geometry in the *Erodium* seed coiling are well-studied, we haven't seen much efforts of applying this design in nature to the engineered systems. Indeed, many plant-inspired soft actuators are available and can exploit carefully designed reinforcing fibers to achieve sophisticated motions. For example, pneumatically pressurized tubes with reinforcing fibers in the *standard* helical pattern have shown pre-programmed twisting and elongation (Li and Wang, 2012, 2015a; Bishop-Moser and Kota, 2015; Connolly et al., 2015, 2017). Hydrogel-based bimorph materials with uniformly distributed fibers or stripes could bend or twist in response to different ambient stimuli (Wan et al., 2018). However, their relatively simple reinforcing fibers are not sufficient to create coiling—a combination of twisting *and* bending—unless we add strain-limiting layers (Polygerinos et al., 2015) or a third fiber (Bishop-Moser and Kota, 2013; Uppalapati and Krishnan, 2018) to the standard helical fibers, or use multiple fluidic chambers (Martinez et al., 2013). These are not as simple and elegant as the tilted helix design in *Erodium*.

Therefore, the objective of this study is to design and test a pneumatically driven soft actuator that can mimic the coiling motion using tilted helical reinforcing fibers. Instead of using the hygroscopic actuation principle like in the *Erodium* plant, we choose pneumatics to ensure a rapid response time, which is crucial for the targeted application of this actuator: soft robotic manipulation. This concept of pneumatic coiling actuator using tilted helical fiber was initially proposed in an earlier paper by the authors (Geer and Li, 2018a), however, it only demonstrated the feasibility obtaining coiling motion without carefully examining the connections between actuator design and corresponding motion. Therefore, this paper particularly focuses on establishing the correlation between the pneumatically-driven coiling motion characteristics and the design of tilted helical fibers. To this end, we fabricate prototypes of the plant-inspired actuator by casting thin-walled elastomeric tubes using 3D printed molds and then wrap Kevlar fibers around them according to the prescribed tilted helical geometry. Experimental validations on these prototypes reveal that the coiling deformation is strongly correlated to the tilt angle. Further parametric analyses based on finite element simulations show that a moderate tilt in the reinforcing helical fiber leads to many coils of small radius, while a significant tilt gives fewer coils of larger radius. Results of this study can offer guidelines for new soft robotic components capable of prescribed coiling motions for object manipulation or field exploration.

The rest of this paper is organized as follows. Section 2 briefly summarizes the design of plant-inspired coiling actuator and how the tilt in helical fiber can generate a combination of bending and twisting. Section 3 details the fabrication and testing of proof-of-concept prototypes. Section 4 discusses the results of a parametric study that elucidates the correlation between tilt helix design and coiling motion characteristics. Finally, section 5 ends this paper with a summary and conclusion.

## 2. Coiling Actuator Design

**Figure 2:**
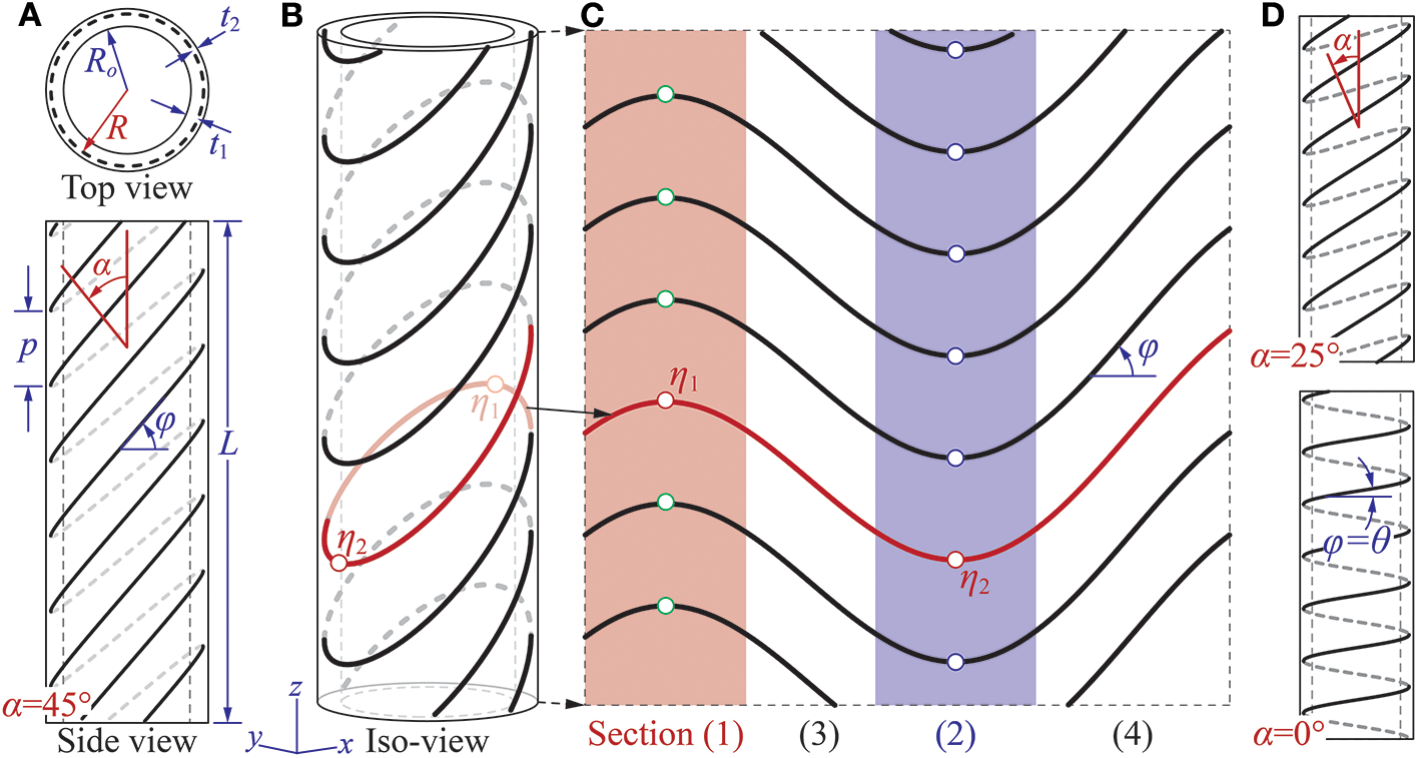
Design of the plant-inspired coiling actuator. **(A)** Top-view and side-view of the actuator showing the multi-layered construction and different design parameters. In this particular illustration, α = 45° and θ = 6°. **(B)** The iso-parametric view. **(C)** The geometry of reinforcing fiber if its cylindrical surface is cut and unwrapped into a flat surface. The four sections discussed in the main text are highlighted. **(D)** Side views of two different actuator designs with two different tilt angles. If α = 0°, the reinforcing fiber has a standard helix shape, so φ = θ according to Equation (5).

The plant-inspired coiling actuator is essentially a pneumatically actuated, thin-walled tube reinforced by tilted helical fibers (Figure 2). The two ends of this tube are sealed and connected to the pressurized air supply. The tube has a total length of *L* and inner radius of *R*_*i*_, and its thin wall—made of highly stretchable elastomeric materials—consists of two layers with thickness *t*_1_ and *t*_2_, respectively. The reinforcing fibers are between these two layers, and their geometry follows the equations

(1) $$\begin{array}{l} x=R\text{ cos }\eta , \end{array}$$

(2) $$\begin{array}{l} y=R\text{ sin }\eta , \end{array}$$

(3) $$\begin{array}{l} z=\frac{p}{2\pi }\eta +A\text{ cos }\eta , \end{array}$$

where *x*, *y*, and *z* are the coordinates of a point on the tilted helix. *R* is the helix radius (*R* = *R*_*o*_ + *t*_1_), *p* is the helix pitch, and η is the parameter representing the helix length. The variable *A* directly defines the tilt angle α of the helix in that

(4) $$\begin{array}{l} \alpha ={\text{ tan }}^{-1}\left(\frac{A}{R}\right). \end{array}$$

If α = 0, the tilted helix becomes a *standard helix* so that *z* = (*pη*)/(2π) (Figure 2D). When the tube has only one family of standard helical fibers, it exhibits a combination of twisting and elongation under internal pressure (Bishop-Moser and Kota, 2015; Connolly et al., 2015, 2017). The magnitude of such twisting is “programmable” by prescribing the ratio between helical radius *R* and pitch *p*. One can also use more complicated helical patterns to enrich the corresponding motion. For example, combining two standard helices with different pitches can offer more freedom for programming the twisting motions. Combining two standard helices of the same pitch but opposite winding directions (aka. one right-handed and the other left-handed) can eliminate twisting so that only elongation or contraction is obtainable (Bishop-Moser and Kota, 2015; Polygerinos et al., 2015; Connolly et al., 2017). The standard helix, however, is fundamentally *axisymmetric* in that it repeats itself after being rotated by any angles along its longitudinal axis. So it is incapable of generating any non-axisymmetric motions like bending unless we intentionally break this axisymmetry.

The introduction of tilt angle α can effectively eliminate the axisymmetry, so it is the crucial design factor that enables the bending and eventually coiling motions. In the standard helix, *z* coordinate increases monotonically as η increases, but this is not true in the tilted helix. By defining φ as the angle between reinforcing fiber and cylinder circumference (Figure 2), one can use trigonometry to show that

(5) $$\begin{array}{l} \text{tan }\varphi =\frac{\partial z}{R\partial \eta }=\frac{p}{2\pi R}-\text{tan }\alpha \text{ sin }\eta =\text{tan }\theta -\text{tan }\alpha \text{ sin }\eta , \end{array}$$

where θ is the fiber angle of the corresponding standard helix. Assuming α > θ, one can then find two sets of critical points (η_1_, η_2_) along the tilted helix by solving φ = 0 in Equation (5) so that

(6) $$\begin{array}{l} \eta_{1}={\text{sin}}^{-1}\left(\frac{p}{2\pi R\text{ tan }\alpha }\right)+2n\pi , \end{array}$$

(7) $$\begin{array}{l} \eta_{2}=-{\text{sin}}^{-1}\left(\frac{p}{2\pi R\text{ tan }\alpha }\right)+\left(2n+1\right)\pi , \end{array}$$

where *n* is an integer. One can then *intuitively* understand how this tilted helical geometry can generate the combination of bending and twisting by dividing it into four sections shown as (1), (2), (3), and (4) in Figure 2C. Section (1) and (2) correspond to η ∈ [η_1_ − π/4η_1_ + π/4] and η ∈ [η_2_ − π/4η_2_ + π/4], respectively. The reinforcing fibers in these two sections are concave or convex curves that are perpendicular to the tube axis at their center points. Thus, one can deduce that these two sections are primarily responsible for generating the bending motion. On the other hand, sections (3) and (4) are between sections (1) and (2), and the reinforcing fibers in these two sections are similar to the standard helical fibers. That is, they show an oblique angle with respect to the tube axis. Thus, one can deduce that these two sections are primarily responsible for generating the twisting motion. The fiber orientations in these four sections change significantly if the tilt angle α changes, so the following experimental study focuses on the correlations between α and the coiling motion.

## 3. Proof-of-Concept Testing

**Figure 3:**
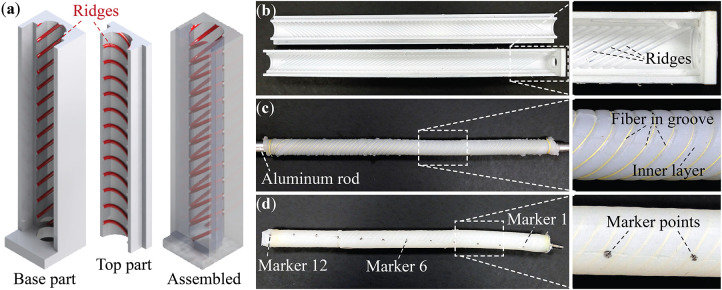
Fabrication of the plant-inspired coiling actuator. **(a)** CAD models of a section of the primary molds. The ridges for fiber placement are highlighted. **(b)** The finished primary molds from 3D printer. **(c)** The assembly consists of an aluminum rod, cured inner layer, and wrapped Kevlar fiber. **(d)** A finished coiling actuator. The end adaptor for pressurized air supply and the markers for displacement measurement are highlighted. Note that for every marker shown in this picture, there is another one on the opposite side behind the actuator body.

To verify that the tilted helix can indeed generate coiling motion by a combination of testing and bending, we fabricate and test three proof-of-concept prototypes of different tilt angles: α = 41, 51, and 61°. The fabrication method is adapted from the previous studies in soft robotic actuators (Polygerinos et al., 2015; Geer and Li, 2018b) and includes three consecutive steps: (1) designing and 3D printing molds, (2) tube casting and fiber wrapping, and (3) creating end caps for pressure sealing (Figure 3).

The first step is to construct two sets of molds according to the coiling actuator design. One is for creating the inner layer shown in Figure 3a, and we refer them as the “primary mold.” The other set of “external molds” are for the outer layer to keep the reinforcing fiber in place. All molds are made in an Object Connect 350 3D Printer using Nylon material. The primary molds have protruding ridges distributed in the tilted helix pattern to create grooves in the casted inner layer (Figures 3b,c). These grooves, which are 0.25 mm in radius, can facilitate the fiber wrapping in the next step.

In the second step, we cast the inner layer of the coiling actuator using the primary molds, an aluminum rod of a 6.4 mm radius, and a vacuum-degassed mixture of DragonSkin 10 Slow silicon rubber (from Smooth-on). After curing at room temperature, we remove the primary molds and manually wrapped the Kevlar fiber (0.035 mm in diameter, from McMaster Carr) along the exposed grooves (Figure 3c). Then we place this assembly into the external molds and cast the outer layer using the same DragonSkin 10 rubber mixture.

In the third and final step, we remove the external molds and aluminum rod from the finished actuator tube, and then dip the tube ends into an uncured rubber mixture for 5–10 min to create the end caps. After curing, we insert a vented screw into one cap and connected it to the pneumatic pressure supply (Figure 3d). Detailed design parameters of these prototypes are in Table 1. It is worth emphasizing that the finished prototypes have two evenly-spaced tilted helix fibers to ensure sufficient fiber coverage.

**Table 1:** Baseline design parameters and material properties of the coiling actuators in experimental study and finite element simulations.

**Parameter**	**Value**
Inner radius (*R*_*o*_)	6.4 mm
Inner layer thickness (*t*_1_)	1.7 mm
Outer layer thickness (*t*_2_)	1.8 mm
Pitch (*p*)	10 mm
Underlying standard helix angle (θ)	11.1°
Tilt angle α	41, 51, and 61°
Total length including end plugs	339 mm
Effective length without end plugs (*L*)	305 mm
Kevlar fiber stiffness (*E*_*f*_)	31.6 GPa
Kevlar fiber Poisson's ratio (ν)	0.36
Odgen model material properties	$\mu_{1}=9.11\times 10^{-4}$ MPa, α_1_ = 5.88
	$\mu_{2}=6.75\times 10^{-2}$ MPa, α_2_ = 1.45

We hang the finished actuator vertically and fix its upper end to a custom made aluminum frame, then use a DC voltage/pressure transducer to pressurize the actuator from 0 to 82.7 kPa with an increment step of 20.7 kPa (Controlair Type 900-ELA E/P pressure transducer and Tenma 72-2690 DC power supply). To measure the actuator deformation accurately, we custom made a motion tracking system by placing two motion depth cameras at the opposite sides of the coiling actuator (Intel Realsense Depth Camera D415). We draw twelve pairs of marker points, labeled as 1–12, on the actuator surface so that each pair lies on the opposite side (Figure 3d). In this way, the motion track cameras can record these maker point positions in 3D space, and the averaged results of these twelve pairs can reflect the motion of the actuator longitudinal axis.

**Figure 4:**
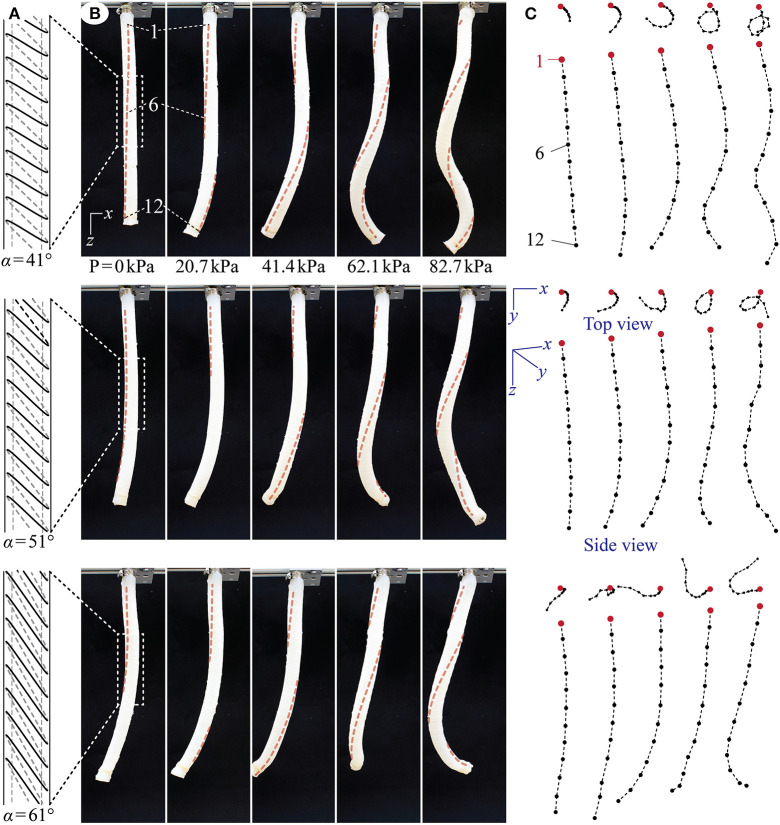
Proof-of-concept test results from the actuator prototypes with α = 41° (top row), 51° (middle row), and 61° (bottom row). **(A)** To-scale schematic drawing of the tilted reinforcing fiber geometry. Only one string of fiber is shown here for visual clarity, but the actuator prototypes each has two strings of evenly spaced fibers to ensure sufficient coverage. **(B)** The actuator prototypes at different pressure levels. To better illustrate the twisting motion in these pictures, we added dashed lines to connect the marker points on the actuator surface. **(C)** The approximated motion of the actuator longitudinal axes based on the depth camera readings. The cameras have limited accuracy, so they generate small and random errors when recording the maker position in the y-axis. Therefore, the center axis can seem non-smooth on some occasions. This error, however, does not hinder us from understanding the overall coiling motion characteristics.

Figure 4 summarizes the deformation of the pressurized coiling actuators (also see the Supplemental Video 1). As the internal pressure increases, all actuator prototypes exhibit a unique “elongate and coil” motion through a combination of elongation, bending, and twisting. Moreover, these actuators primarily show elongation and bending at low pressure, and when the pressure increases beyond a threshold value (~41 kPa), twisting emerges rapidly and drives the actuator bodies into different coil shapes. This non-uniform appearance of various deformations reflects the non-linear elastic properties of DragonSkin silicon rubber.

Moreover, by comparing the deformations between different actuator prototypes, one can observe a strong influence of the tilt angle on the coiling motion. If the title angle α is small, the actuator deforms into a thin coil with a relatively small radius. On the other hand, if the tilt angle α is large, the actuator deforms with a relatively larger coiling radius. Since the actuator length is the same, the coiling radius is inversely related to the number of completed coils. That is, while the actuator prototype with α = 41° manages to complete more than one and a half coils, the one with α = 61° can only achieve about a half coil.

It is worthwhile to discuss the differences between the coiling motion exhibited by these engineered actuators and the motion of Erodium awn tissues shown in Figure 1. Firstly, although these two systems have similar tilted helical reinforcing fiber design, they differ in the actuation principles. The plant tissue achieves coiling by hygroscopic shrinking, while the engineered actuators achieve coiling by pneumatic expansion. As a result, their motion characteristics have some differences. In particular, the longitudinal elongation shown by the coiling actuators is the result of the pneumatic pressurization, but the Erodium tissue does not exhibit any significant elongation or contraction. Secondly, the scaling between the engineered actuator and Erodium tissues are quite different. That is, the plant tissue has much slender shape. If we reduce the radius of the pneumatic actuator, we would likely observe a more prominent bending component like in the plant tissues because of the smaller bending stiffness. However, the correlations between the actuator radius, length, and title fiber design are strongly non-linear, as indicated by relevant studies on the plant tissue (Aharoni et al., 2012). We chose the scale of these pneumatic actuators to ensure manufacturability based on the 3D-printed mold and manual fiber wrapping method.

## 4. The Influences of Tilted Helix Geometry on Coiling Motion

To further understand the influence of tilted helical fiber geometry on coiling motion, we conduct two finite element simulations using ABAQUS^TM^. The first simulation aims to validate the correlation between tilt angle and coiling observed in the experiments, while the second simulation explores the tilted helix design space more comprehensively.

In the first simulation, we construct finite element models according to the prototype design parameters in Table 1, and hung the actuator vertically by applying fixed boundary conditions at its upper end. Quadratic and tetrahedral elements (C3D10H) are used to mesh the two layers of the actuator wall separately, and the hybrid formulation is adopted to avoid volume locking since the silicon rubber material is assumed incompressible. We mesh the reinforcing fibers by quadratic beam elements (B32), apply tie constraints between the fiber and inner layer, and then merge the outer layer to the inner layer.

**Figure 5:**
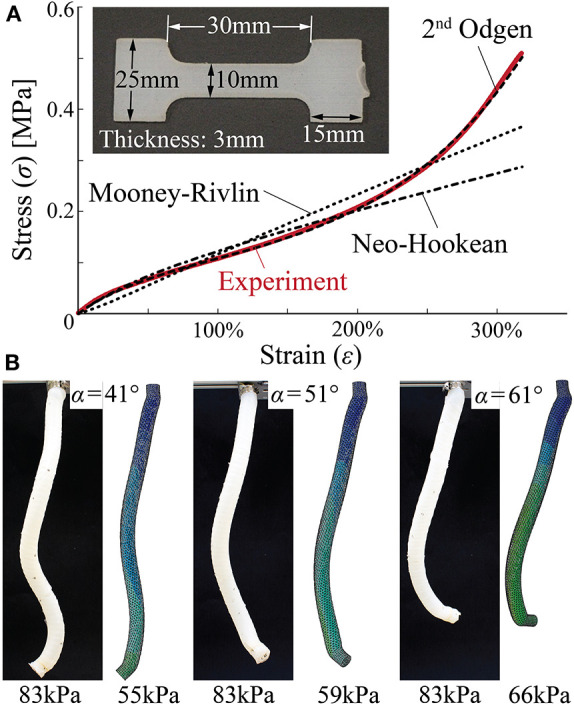
Set up of finite element simulations. **(A)** Testing dogbone samples to model the hyperelastic properties of DragonSkin 10 rubber material. Compared to the Neo-Hookean and Mooney-Rivlin models, second-order Ogden model can accurately reproduce the nominal stress-strain relationship from the uniaxial tensile test. **(B)** Comparing finite element simulation results and proof-of-concept test results.

The DragonSkin 10 silicon rubber used for constructing the actuator wall is strongly hyperelastic, so it is challenging to obtain an accurate description of its non-linear elastic properties. To this end, we cast a dogbone specimen using 3D printed molds (Figure 5A) and measure its reaction force under stretch on a universal testing machine (Instron 1125, 325% of maximum strain). We fit different hyperelastic material models to the measured nominal stress-strain relationship, and the second-order Odgen model can give the best agreement throughout the deformation range (Figure 5). The fitted material properties, together with the elastic properties of the Kevlar fiber, are summarized in Table 1.

**Figure 6:**
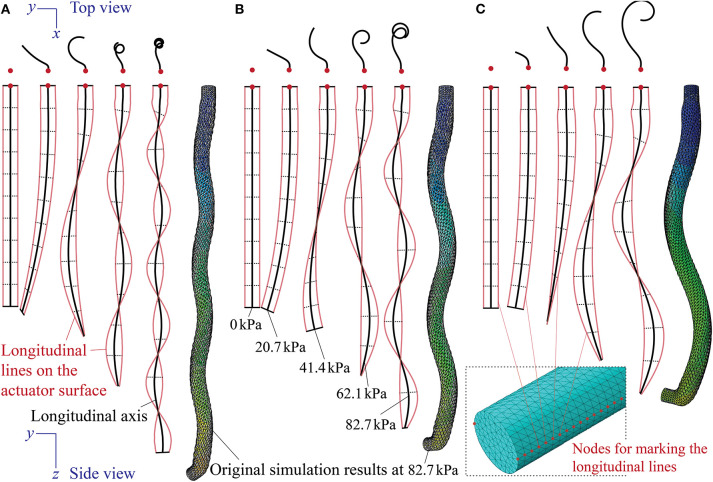
Finite element simulation of the coiling motions at different pressures from actuators with **(A)** α = 41°, **(B)** α = 51°, and **(C)** α = 61°. Designs of these actuators are the same as the physical prototypes described in Table 1. To trace the deformation of longitudinal lines on the surface, we selected two lines of surface nodes on the opposite sides of the actuator (one of them highlighted in the insert figure) and only showed their displacements at different pressures. The longitudinal axis thus is the averaged displacements of these surface nodes.

The finite element models are able to reproduce the elongation and coiling motions observed in the proof-of-concept tests. That is, the actuator deformations predicted by the numerical simulations agree with the experimental observation well (Figure 5B). However, these agreements occur at different pressures in that numerical simulations predicted smaller pressures to achieve similar coiling. There can be a few causes for this discrepancy. The first probable cause is errors in the property modeling of DragonSkin 10 material. Fitting the uniaxial test data alone cannot guarantee the accuracy of the second-order Odgen model, and one can significantly improve the model by using additional tests, such as a bi-axial stretch test. However, these further tests require sophisticated equipment that are not available to the authors. The second probable cause is the fabrication imperfections. Since the actuator prototype assembly is manual, many defects can occur like reinforcing fiber misalignment, entrapped air bubbles in the DragonSkin rubber, and residual stress from removing the aluminum rod that caused the prototype to bend slightly even without pressure. Despite the quantitative differences, the numerical simulations confirm the experimentally-observed correlation between tilt angle and coiling motion. That is, a small tilt angle α generates a significant elongation and small coiling radius so that the actuator can complete a relatively large number of coils. While a large tilt angle gives less elongation and larger coiling radius, so a relatively smaller amount of coils are achieved (Figure 6).

**Figure 7:**
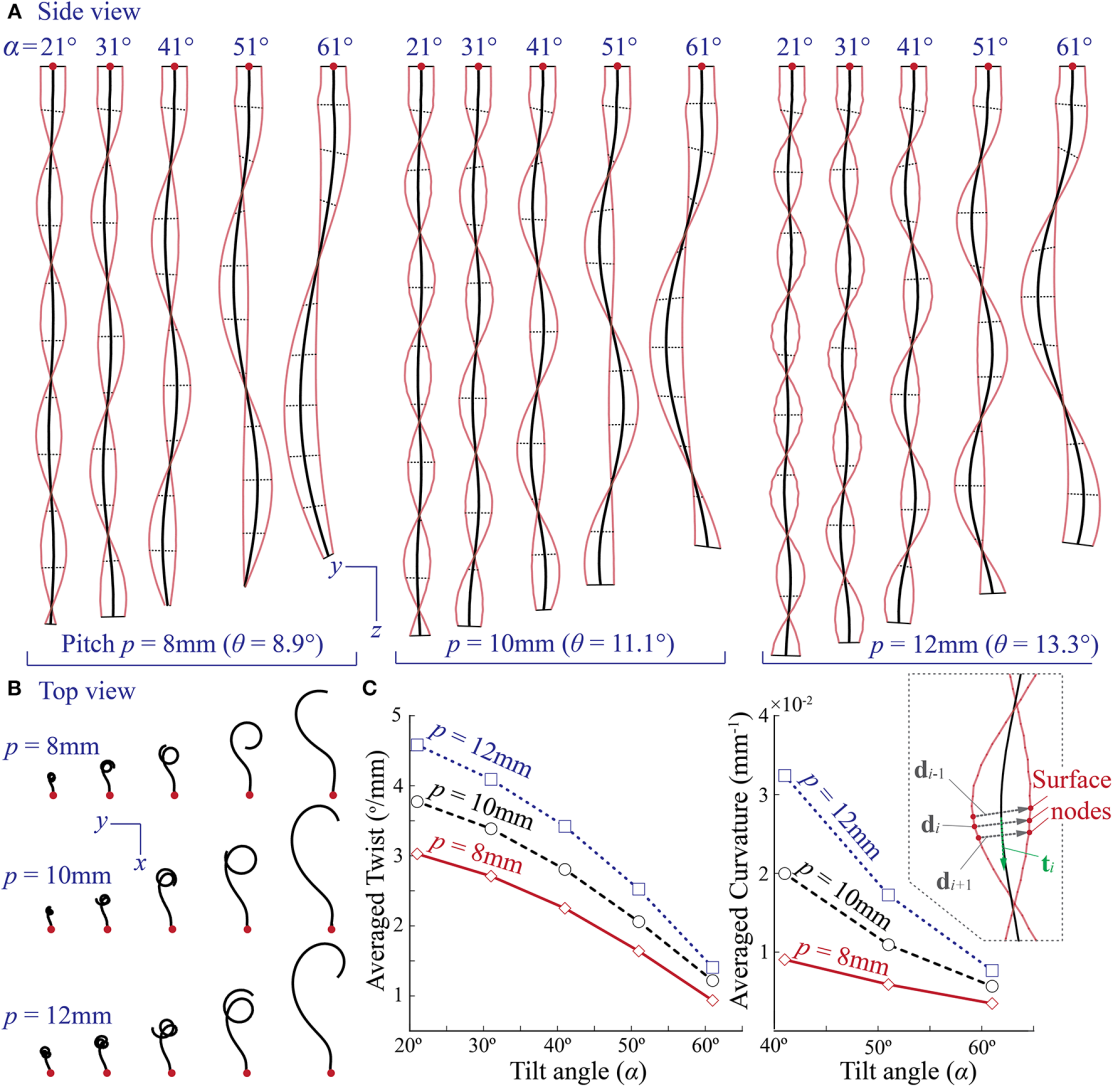
Design parametric study using finite element simulations. **(A,B)** Simulated external shapes of different coiling actuators with various combinations of reinforcing helix tilt angle (α) and pitch (*p*). All of them are subjected to the same internal pressure at 69 kPa. **(C)** The correlation between the averaged actuator twist, curvature, and tilted helix design parameters based on FEA results. The insert figure on the right illustrates the method of calculating local twist and curvature.

By using the finite element model, we conduct a parametric study to further examine the relationship between the tilted reinforcing helix design and coiling motion. In this study, we simulate the actuator deformations based on different combinations of tilt angles (α = 21, 31, 41, 51, and 61°) and pitches (*p* = 8, 10, and 12 mm), and Figure 7 summarizes the results when the internal pressure is 69 kPa. All other actuator design parameters and material properties remain the same as those listed in Table 1. Here, the range of title angles and pitches are chosen carefully to ensure manufacturability. We find that when the tilt angle is bigger than 61°, it becomes quite challenging to wrap the reinforcing Kevlar fiber into the grooves of the actuator inner layer. A pitch bigger than 12 mm would leave too much space between the adjacent fibers (i.e., low fiber coverage); as a result, the rubber-like DragonSkin material between the fibers can bulge under pressure, leading to excessive and localized deformation. It is worth emphasizing that all of the actuators have two strings of evenly spaced reinforcing fibers to ensure sufficient coverage.

The simulation results, shown in Figure 7, reveals that the experimentally observed correlation between tilt angle and coiling motion applies to different pitches. That is, for the same pitch, the actuator can complete several coils of small radius if the tilt angle is low, but fewer coils of larger radius as the title angle increases. We further examined this trend by comparing the averaged twist and curvature of different actuators. Here, the twist refers to rotation of the cross-sections of the coiling actuator about its longitudinal axis. Denote **d**_*i*_ and **d**_*i*+1_ as the adjacent diameter vectors, each defined by two surface nodes on the opposite side of the deformed actuator (insert in Figure 7C). The local twist is

(8) $$\begin{array}{l} \tau_{i}=\frac{{\text{cos}}^{-1}\left({\text{d}}_{i}\cdot {\text{d}}_{i+1}\right)}{\text{d}\eta }, \end{array}$$

where dη is the distance between these two diameter vectors at the *initially undeformed configuration*. The localized curvature is,

(9) $$\begin{array}{l} \kappa_{i}=\frac{\left\|{\text{t}}_{i}^{\prime }\times {\text{t}}_{i}^{\prime \prime }\right\|}{\|{\text{t}}_{i}^{\prime }{\|}^{3}}, \end{array}$$

where **t**_*i*_ is the tangent vector of the deformed longitudinal axis (insert in Figure 7C), and “′” is the derivative with respect to the initially undeformed configuration. Then, the local twist and curvature of the center half of the deformed actuator are averaged to avoid the boundary effects from the two end plugs.

The averaged results, shown in Figure 7C, indicate that both the twist and curvature decrease as the tilt angle α increases. Moreover, a larger pitch seems to amplify both twist and curvature; as a result, the actuator with a larger pitch can complete more coils.

## 5. Summary and Discussion

Inspired by the sophisticated motions shown in the awn of *Erodium* plant seeds, we examine pneumatic actuators capable of generating coiling motion—a combination of twisting and bending. These actuators are essentially thin-walled elastomeric tubes reinforced by tilted helical fibers, which closely resemble the cellulose fiber distributions in the *Erodium* seed awn. However, instead of operating based on the hygroscopic principle like in plants, these actuators use pneumatics to ensure fast response time so that they are suitable for robotic applications. Several actuator prototypes of different fiber tilt angles are fabricated using 3D printed molds, and they manage to achieve coiling. The magnitude of helical tilt angle plays a crucial role in creating the coiling motion because this helical tilt causes the actuator to bend, which is not possible from the standard helical reinforcing fiber. Experimental results reveal that actuators with a smaller tilt angle show significant elongation and can achieve several coils of small radius; while those with a large tilt angle typically make fewer, large radius coils. We also construct finite element models to simulate the coiling. The numerical simulations confirm the correlation between tilt angle and coiling motion characteristics despite the discrepancies in terms of motion magnitude. With this finite element model, we are able to conduct a more comprehensive parametric study by combining different helical tilt angles and pitches. We find that the experimentally observed correlation between tilt angle and coiling applies to different helical pitches, and increasing this pitch would amplify the actuator twisting, leading to more coils of smaller radius. Therefore, one can effectively program the coiling characteristics by carefully designing the reinforcing tilted helix geometry.

**Figure 8:**
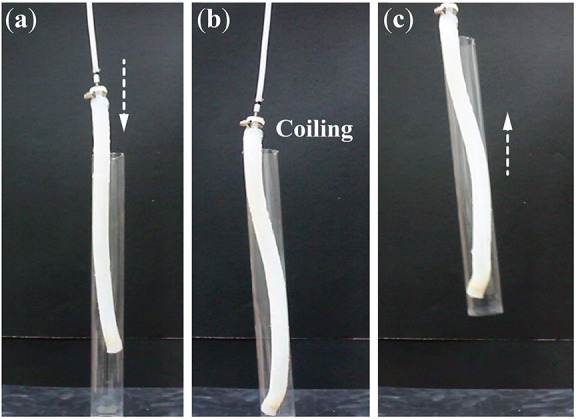
A demonstration of a robotic application of lifting a pipe via coiling from inside. **(a)** The unpressurized actuator is first placed inside a hollow plastic pipe. **(b)** Then, it coils under pressure (21 kPa) and creates contact on the inner pipe surface. **(c)** The pipe is lift up with the help of friction force. The coiling actuator here has a title angle α = 41°.

The elongation and coiling motion exhibited by these plant-inspired actuators are unique and can open up new capabilities in soft robotic manipulation. For example, coiling can be used to manipulate long and slender shaped objects better than simple twisting or bending actuators (like from inside the pipe shown in (Figure 8 and Supplemental Video 2). So these actuators might have potential applications in bio-medical applications like assistive care or rehabilitation. One could also possibility use these actuators to achieve drilling into porous media just like the Erodium tissue, so that we can use these actuators for field exploration or environment monitoring. The results of this study could provide physical insights and a design guideline for future implementations.

## Data Availability Statement

The datasets generated for this study are available on request to the corresponding author.

## Author Contributions

RG and SL conceived the research topic. RG conducted the fabrication, experimentation, and finite element simulation. SL conducted the data analysis and presentation. SI assisted the experiment design and data acquisition. All contributed to the manuscript draft.

### Conflict of Interest

The authors declare that the research was conducted in the absence of any commercial or financial relationships that could be construed as a potential conflict of interest.

## Supplementary Material

Supplemental Video 1 Coiling motion of the plant-inspired actuators with different helix tilt angles.

Supplemental Video 2 A demonstration of slender object handling using coiling motion.
